# Synergy Repetition Training versus Task Repetition Training in Acquiring New Skill

**DOI:** 10.3389/fbioe.2017.00009

**Published:** 2017-02-27

**Authors:** Vrajeshri Patel, Jamie Craig, Michelle Schumacher, Martin K. Burns, Ionut Florescu, Ramana Vinjamuri

**Affiliations:** ^1^Sensorimotor Control Laboratory, Department of Biomedical Engineering, Chemistry, and Biological Sciences, Stevens Institute of Technology, Hoboken, NJ, USA

**Keywords:** kinematic synergies, hand, motor learning, principal component analysis, rehabilitation

## Abstract

Traditionally, repetitive practice of a task is used to learn a new skill, exhibiting as immediately improved performance. Research suggests, however, that a more experience-based rather than exposure-based training protocol may allow for better transference of the skill to related tasks. In synergy-based motor control theory, fundamental motor skills, such as hand grasping, are represented with a synergy subspace that captures essential motor patterns. In this study, we propose that motor-skill learning through synergy-based mechanisms may provide advantages over traditional task repetition learning. A new task was designed to highlight the range of motion and dexterity of the human hand. Two separate training strategies were tested in healthy subjects: task repetition training and synergy training versus a control. All three groups showed improvements when retested on the same task. When tested on a similar, but different set of tasks, only the synergy group showed improvements in accuracy (9.27% increase) compared to the repetition (3.24% decline) and control (3.22% decline) groups. A kinematic analysis revealed that although joint angular peak velocities decreased, timing benefits stemmed from the initial feed-forward portion of the task (reaction time). Accuracy improvements may have derived from general improved coordination among the four involved fingers. These preliminary results warrant further investigation of synergy-based motor training in healthy individuals, as well as in individuals undergoing hand-based rehabilitative therapy.

## Introduction

From learning to grasp a ball to learning to type at a keyboard, we are continuously tasked with acquiring new motor skills throughout life. There is a dynamic combination of both cognitive (strategy formation and task comprehension) processes and motor processes (feedback integration and motor execution) that allow us to learn and execute these motor skills. In the 1940s, Nicholais Bernstein provided an intriguing definition of a motor skill: “… not a movement formula … [but] an ability to solve one or another type of motor problems” (Latash and Latash, 1994). Subsequently, motor-skill learning can be defined as “a set of processes associated with practice or experience leading to relatively permanent changes in the capability for responding” (Schmidt, 1976). In these definitions, an emphasis is placed on learning a response through practice rather than memorizing motor patterns. Much research has been dedicated to determining what phenomena occur during practice and how these phenomena lead to a permanently acquired motor skill.

It has long been known that repetition is essential to learning a new skill, or procedural learning. Skill learning theories suggest that motor skills are initially developed in a fast cognitive-based stage. During this time, explicit learning is based upon declarative knowledge and working memory. At the neuronal level, we see decreased inhibition allowing for increased excitability in the primary motor cortex (M1) (Karni et al., 1995). Simultaneously, structural changes such as increased myelination (Sampaio-Baptista et al., 2013) and clustering of new dendritic spines (Fu et al., 2012) occur in various relevant pathways including multiple M1 layers (Huber et al., 2012), sensorimotor cortex (Sampaio-Baptista et al., 2013), and cerebellum (Cantarero et al., 2015). These changes support the ascension from the cognitive phase to the associative phase, where ineffective actions are filtered out. Individuals become unconsciously sensitive to regularity and other implicit learning mechanisms. After extensive practice, the autonomous stage is reached; retention of the procedural memory of the motor skill is thought to be stored in cortical–striatal circuits (Doyon and Benali, 2005).

Repetition training is often compared to interleaved training, which involves a more varied protocol. Interleaved training may engage more prefrontal and parietal regions because each task requires the individual to reconfigure motor commands (Li and Wright, 2000; Kantak et al., 2010). Repetition training versus interleaved training, however, allows for greater M1 excitability. It has been found that interleaved training performs worse than repetition training in short-term performance, but better in long-term performance transference (Dromerick et al., 2009). The varied presentation may promote implicit learning because it provides greater exposure to correlated variables (Meier and Cock, 2010). It is apparent that both repetition and interleaved practice have advantages and disadvantages; consequently, balancing both learning strategies may allow us to optimize motor-skill learning.

This balance of these learning variables is especially important for individuals in rehabilitative therapy that must relearn essential skills, with limited time and usually, limited muscle strength. Because the individuals are relearning previously acquired motor skills, researchers have turned to natural motor control and motor learning concepts to determine ways of optimizing physical therapy strategies (Krakauer, 2006. Muratori et al., 2013). For example, task-orientated training with spaced practice versus conventional massed practice (learning with short or no intervals) may promote long-term memory of the learned motor skills (Dromerick et al., 2009). The ability for implicit and explicit learning in individuals with stroke has also been studied. Depending on the location of cerebral damage, implicit learning may be compromised (Ackermann et al., 1996; de Guise et al., 1999), and explicit information may be detrimental (Boyd and Winstein, 2003). This suggests that skills normally learned *via* implicit and explicit mechanisms need to be presented in a different format.

In this study, we propose a new mechanism of motor training: synergy training. In its most general definition, synergies represent learned motor primitives that reduce the computational burden of the central nervous system (CNS). For example, throughout life, the hand’s ability to dexterously grasp and manipulate objects found in activities of daily living is a skill that requires control over 30 degrees of freedom. It is hypothesized that after years of learning, the motor control system has optimized the “reach and grasp” motor task. This skill may be represented in the CNS as motor synergies that encode simultaneous coordination of the many involved joints versus individual control of each joint. Using dimensionality reduction techniques, we (Vinjamuri et al., 2010; Patel et al., 2015; Burns et al., 2017) and others have characterized this synergy subspace at various hierarchical levels including neural (Saleh et al., 2010), muscle (d’Avella et al., 2011), and kinematic (Santello et al., 2002; Vinjamuri et al., 2014). If synergies represent motor strategies that have been optimized through experience (involving both explicit and implicit learning mechanisms), they may useful during the learning experience itself. Furthermore, continued advancements in robotic technologies will soon allow therapist to implement synergy training in individuals who have altered synergies, poststroke (Cirstea and Levin, 2000; Michaelsen et al., 2001; Roby-Brami et al., 2003; Zackowski et al., 2004; Neckel et al., 2006; Roh et al., 2013; Jarrassé et al., 2014).

In this study, we model the potential effects of synergy-based training using a simplified motor-skill learning framework. In order to keep the study related to hand motor skill, we design a new task that requires users to coordinate control of joints in the four fingers. We compared traditional task repetition training with a new synergy-training protocol to determine the effects of each method. Subjects are tested after a specific training procedure to measure retention. Additionally, they are then tested on a separate set of tasks to measure transference. We hypothesize that the task repetition group will exhibit stronger performance during the retest phase, while the synergy group will exhibit stronger performance during the transference phase.

## Materials and Methods

### Overview

For this study, 16 right-handed healthy individuals were recruited (10 males, 6 females, mean age 21.5 ± 1.5) under Stevens Institute of Technology Institutional Review Board approval. Through self-report, subjects that were mildly skilled musicians were excluded from the study. Subjects were assigned to one of three groups: task repetition training, synergy training, and control. Data from one subject (subject 2) were removed because of data collection complications, resulting in five subjects in each group.

### Experimental Procedure

**Figure 1:**
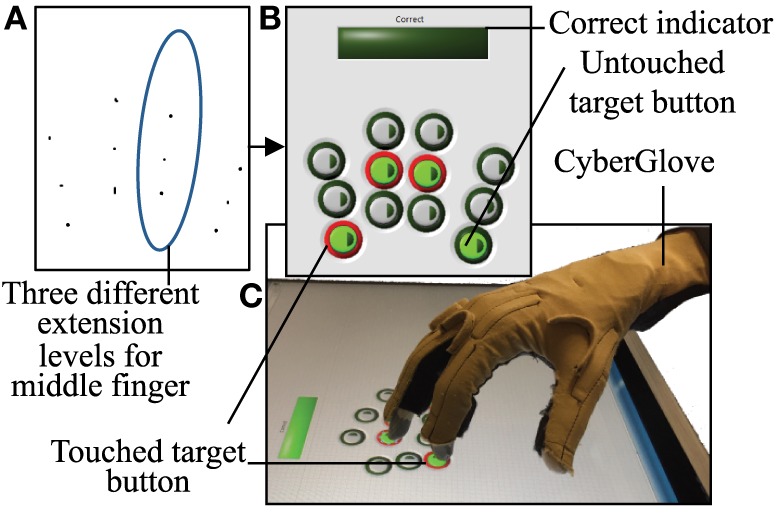
**A 3 × 4 button grid is used to accomplish the touch task**. **(A)** In order to obtain button positions that align with each subject natural axis of extension, touch points at three different extension ranges were recorded. **(B)** The touch task involves touching four buttons that have been lit green after an audio start cue. If a button is touched, it is lit red. If all four buttons are correctly touched, a “Correct” bar above the grid is lit green. **(C)** CyberGlove is used to record joint angles during the experiment.

A user interface was created in LabVIEW 2014 (National Instruments Corporation, Austin, TX, USA) using Touchscreen Toolkit (Aledyne Engineering, Morgan Hill, CA, USA). The interface was displayed on a touchscreen monitor with multi-touch compatibility (Acer, San Jose, CA, USA). As seen in Figure 1, a 3 × 4 button grid is displayed on the touchscreen. Each column, from left to right, corresponds to the index, middle, ring, and pinky. Three rows represent three ranges of extensions that each finger will have to achieve. The alignments of these buttons were subject based. Before testing began, the subject was seated in the experimental setup (wrist strapped down, which is further described below). Starting in a closed fist position, the subject was asked to extend to three comfortable levels. The first level requires that the subject has enough extension so that either the fingertip or finger pad makes contact with the screen. The third level requires the subject to extend as much as possible, while still making contact with the screen. The second level fell between these two levels. Touch points (Figure 1A) collected and imported into MATLAB (MathWorks, Natick, MA, USA). Pixel locations of these touchpoints determined button locations (after minor adjustments to account for button size), which were then imported by LabVIEW (Figure 1B).

A single touch task requires subjects to touch the four buttons that are lit. Out of a total of 81 possible button combinations; four button combinations were removed due to anatomical difficulty in achieving the postures. The remaining 77 combinations were randomly divided into two sets: set 1 consisted of 45 button combinations and set 2 consisted of 32 button combinations. The procedure for a single task is as follows: subjects started with hands in a closed fist position. An audio start cue coincided with four target buttons lit green. An audio stop cue was given after either four buttons were touched (incorrect or correct) or 4 s had passed. During the task, if a button was touched, the outer rim of the button turns red (Figure 1B). If all four target buttons are correctly touched, a “correct bar” lights green. Between each task, a 3.5 s break is given, during which the subject returns his/her hand to a fist position. Subjects were instructed to complete each task as “accurate” and “simultaneous” as possible. Accuracy pertains to pressing the four correct buttons and simultaneous pertains to pressing each button simultaneously versus sequentially. In order to keep the task confined to finger movements only, the wrist was strapped to a board positioned above the touchscreen.

#### Phase 1

**Figure 2:**
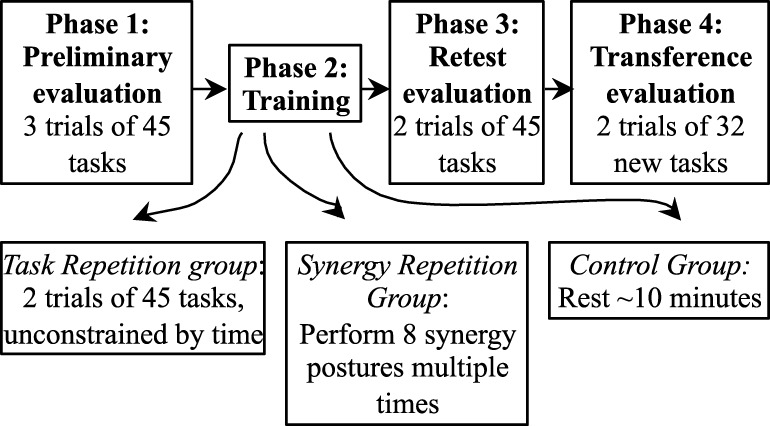
**The experiment consists of four phases**. In Phase 1, subjects perform 3 trials of 45 tasks. Phase 2 involves different forms of training. Subjects in the task repetition group repeat the same 45 tasks twice and with extra time. If subjects are in the synergy repetition group, eight postural synergies were derived from kinematic data in Phase 1. These subjects trained on performing the eight postural synergies. Subjects in the control group were required to rest for ~10 min. In Phase 3, all subjects were retested on the tasks performed in Phase 1. In Phase 4, 32 new tasks were introduced to test transference of the new skill.

The experimental procedure (Figure 2) consisted of four phases: Phase 1—preliminary evaluation, Phase 2—training, Phase 3—retest evaluation, and Phase 4—transference evaluation. In Phase 1 (preliminary evaluation), subjects performed a total of three trials. Each trial consisted of performing the 45 tasks in set 1 and lasted a maximum of ~6 min. An optional 1 min break was given between trials. The first trial allowed subjects to familiarize themselves with the task and task procedure; therefore, data from trial 1 were discarded. Trials 2 and 3 were used to determine baseline performance for each subject.

#### Phase 2

**Figure 3:**
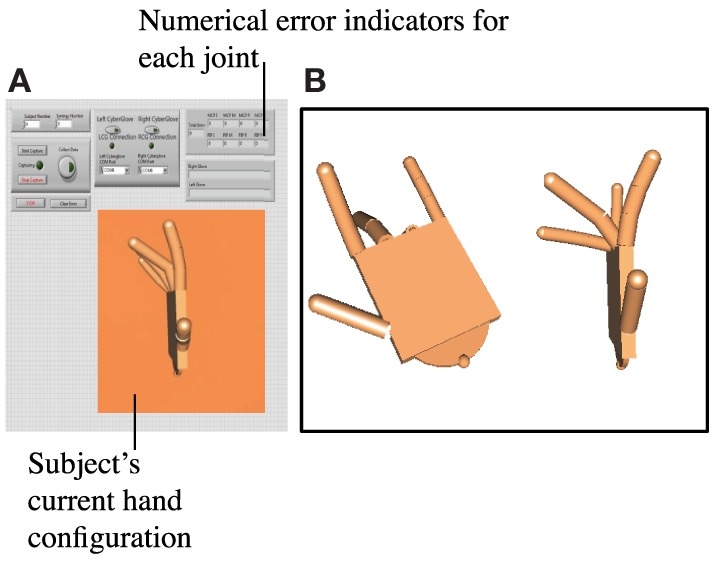
**(A)** A separate program was used for synergy repetition training. The program imitated the hand’s current configuration using a custom virtual hand model. Numerical indicators showed the error between current and target synergy postures at each joint. Subjects were told if a joint fell outside of the 20° error range. **(B)** On a second monitor, the target synergy posture was shown. Two views were given (a top angled view and a side view) so that subjects were able to imitate each of the eight joints.

In Phase 2 (training phase), subjects were trained according to their assigned group. Subjects in the task repetition training group performed 2 additional trials of the same 45 tasks from set 1, but without the time constraint (timeout of each task was set to 8 s, and intertask breaks were set to 6 s) and wrist constraint. Subjects were told to use this phase and the extra time to improve their accuracy and simultaneity. Subjects that were assigned to the synergy-training group practiced eight postural synergies derived from Phase 1, trials 1 and 2. Postural synergy derivation and synergy training is described in detail in Section “Derivation of Postural Synergies and Synergy Training”; a brief description is provided here. In synergy training, subjects were first familiarized with each of the 10 derived synergy postures, which were displayed on a computer screen (Figure 3). Still wearing the CyberGlove, the subject practiced performing each posture until a minimum error between the hand posture and the displayed synergy posture was reached (after approximately two to three attempts). Then, starting in a closed fist position, each posture was quickly performed for three repetitions (each posture was queued by displaying the synergy posture on the screen). The goal of this training was to have subjects become comfortable and familiar enough with each synergy posture that they are able to rapidly perform it when queued. Additionally, subjects were explicitly told that these postures were to be learned as much as possible to “help” improve task performance. We attempted to keep training procedures for both the task repetition training and synergy-training groups as even as possible by implementing the following procedures. First, the maximum time allowed for the training phase was kept to 20 min. During this time, the task repetition training group performed ~90 postures, and the synergy-training group performed ~60 postures. Second, the following feedback was implemented in both groups. For the task repetition group, just as in Phase 1, the red button outlines indicated when buttons were touched, and a green bar (“Correct Indicator”) indicated when tasks were correctly performed; for the synergy group, numerical values indicated which joints were not adequately similar to those of the displayed synergy posture. Joint angles within a 20° error range were considered acceptable. Third, both groups maintained creating postures from a closed fist position. Fourth, the time constraint was removed, so that subjects could focus on the creation of each posture. Fifth, because the wrist needed to be free while learning synergy postures, in both training groups, a wrist constraint was not used. Subjects that were assigned to the control group rested for ~10 min during the Phase 2.

#### Phase 3 and Phase 4

In Phase 3, subjects were retested with 2 trials of the same 45 tasks from set 1. This phase was used to determine improvements from baseline evaluation. In Phase 4, 32 additional tasks (set 2) were introduced. Two trials were performed. These trials were used to test the transference of motor skills gained from Phases 1–3 to similar, but untested tasks.

**Figure 4:**
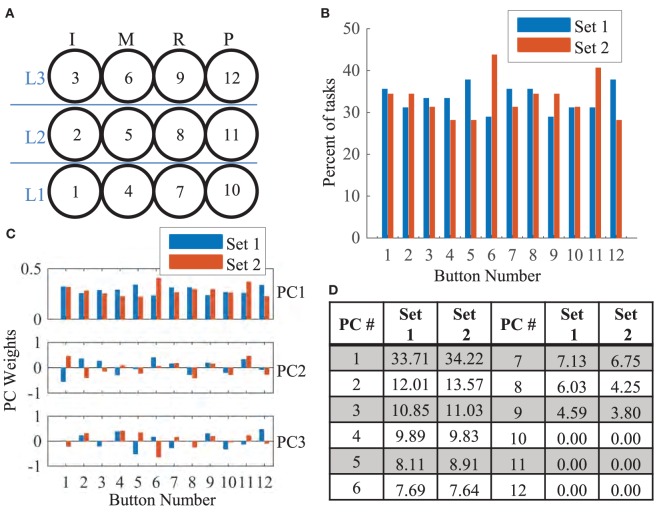
**(A)** The 3 × 4 button grid required three levels of extension (labeled in blue) for each finger [index (I), middle (M), ring (R), and pinky (P)]. For reference, each button number is provided in black. **(B)** The percent number of tasks that involved a specific button is shown for set 1 (blue) and set 2 (red). Buttons 5, 6, 11, and 12 showed differences in frequency. **(C)** The first 3 (of 12) principal components derived from set 1 and set 2 tasks are shown. In PC1, weight proportions are similar to **(B)**. In the remaining PCs, the covariance patterns are distributed. **(D)** The fraction of variance that each of the 12 PCs account for is shown. Distributions of variance are similar across both groups.

To compare the similarity of the 45 tasks in set 1 and the 32 tasks in set 2, we looked at the overall frequency in which each button was pressed as well as the general patterns found in each group. Figure 4A shows each of the 12 buttons numbered in black. Figure 4B shows the percent of tasks that involved each button. Buttons 5 and 12 were proportionately used more often in set 1 tasks while buttons 6 and 11 were proportionately used more often in set 2 tasks. Principal component analysis (PCA) was then used to determine if the overall required patterns are statistically similar between set 1 and set 2. Of the 12 total principal components derived, the first three are shown in Figure 4C. Statistically, the pattern described from Figure 4B and seen in PC1 of Figure 4C accounts for ~34% of the total variance (Figure 4D) for both groups. PCs 2–9 account for the remaining variance. Thus, the majority of the variance is scattered equally across the 12 buttons; however, there is a slight skew toward buttons 5 and 12 in set 1 tasks and buttons 6 and 11 in set 2 tasks. This is further discussed in Section “Discussion.”

### Data Collection

A right-handed CyberGlove (CyberGlove Systems LLC, San Jose, CA, USA) equipped with 18 sensors was used to capture hand movements during the experiment at a rate of 125 Hz. In this study, only data from the metacarpophalangeal (MCP) and proximal interphalangeal (PIP) joints of the four fingers were used. The glove was calibrated for each subject using custom goniometers ranging from −10° to 90°. Once the glove was dawned, it was not removed until the study was completed. Subjects also wore a glove liner that was coated with a liquid allowing the fabric to be touchscreen compatible. CyberGlove data were recorded with the same LabVIEW program that controlled the task. Additionally, for each task, the identity of the buttons that were pressed and the task completion time (CT) were recorded.

### Derivation of Postural Synergies and Synergy Training

In this study, we hypothesized that synergy-based training provides a means of reinforcing spatial joint patterns that generalize to a large range of tasks. In order to determine these movement patterns, we used PCA, a commonly used dimensionality reduction technique for synergy derivation (Santello et al., 2002; Thakur et al., 2008; Vinjamuri et al., 2010). End postures taken from Phase 1, trials 1 and 2, provides joint configurations for 45 tasks, with 2 repetitions each. The mean posture across both repetitions was used to create an *m* × *n* joint angle matrix (*J*), where *m* is the number of tasks (*m* = 45) and *n* is the number of joints (*n* = 8). Singular value decomposition is used to approximate *J* such that:

(1) $$J=U\sum R^{\prime },$$

where orthogonal *U* (*m* × *m*) contains left singular vectors, orthogonal *R* (*n* × *n*) contains right singular vectors, and Σ (*m* × *n*) contains the square root of singular values in its diagonal. The rows of *R*′ contain eigenvectors of *J*′*J*, or principal components. These eight principal components are considered synergies. Therefore, we have a total of *s* = 8 synergies. Importantly, although these synergies were derived from only a subset of all possible tasks (Phase 4 tasks are not included), they each emphasize specific joint patterns that can then be combined to produce new postures.

After synergies were derived, each synergy vector was multiplied by a maximum possible weight such that the joint angles still fell within the range of normal movement [set from −10° to 90° for MCP joints and 0° to 90° for proximal interphalangeal (PIP) joints]. A separate LabVIEW program was used to display the resulting synergy postures. A virtual hand model (developed using the Simulink 3D Animation toolbox from MATLAB) showed the target synergy posture. As seen in Figure 3, top and side views were provided. Numerical indicators showed the target angle for each joint as well as the subject’s current joint angle. The synergy training procedure involved the following steps:

(1) Target synergy 1 posture is displayed to the subject. The subject attempts to perform this posture. The LabVIEW program calculates the error between the target posture and the subject’s attempted posture. The subject then reattempts the posture. Approximately two to three attempts were usually required for the subject to create the synergy posture with error below 20° at all joints. This is repeated for all 10 synergies.
(2) The subject is queued through all 10 synergy postures quickly. Upon seeing a synergy posture, he/she creates the matching hand configuration quickly. The experimenter then queues the next posture. This was repeated for a total of three rounds.

### Reconstruction

In order to determine how subjects may have recruited synergies in Phases 3 and 4, the end posture of each task was reconstructed using weighted synergies. Let *w* (1 × *s*) represents the weight of each synergy and *S* represents the synergy matrix, which is equal to *R*′. Each end posture is represented by joint angles contained in *A* (*n* × 1). The following optimization problem (Vinjamuri et al., 2010) was used in the selection of synergies and weights:

(2) $$\text{Minimize}||w{||}_{1}+\frac{1}{\lambda }||wS-A{||}_{2}^{2},$$

||·||_1_ represents the *l*_1_ norm, allowing minimization of recruited synergies, ||·||_2_ represents the *l*_2_ norm or Euclidian norm of a vector, minimizing error between reconstructed and target posture, and λ is a regulation parameter calculated equal to 0.01 × (max(abs(2 × *A* × *S*′))) (Koh et al., 2007; Vinjamuri et al., 2010). Reconstruction error (RE) is considered a measure of synergy usage because it shows how well task end postures imitated weighted and combined synergy postures.

### Data Analysis

Task-related variables were compared across phases (Phase 1—preliminary evaluation, Phase 3—retest evaluation, and Phase 4—transference evaluation) and across the three groups—task repetition, synergy, and control. Data from the two trials in each phase are accumulated.

To measure acquired motor skill in each phase, the percent correct (PC) and their average CT were measured. Angular data recorded from eight sensors on the data glove were filtered with a 5 Hz with a low-pass Butterworth filter. Various variables were used to detect any training-related kinematic changes. This includes reaction time (RT), peak velocity (PV), time of peak velocity (tPV), and overextension (OE). For each task, RT is defined as the first time one of the eight joints reaches 1% of PV. In each task, the magnitude and time at which PV occurs in each joint were recorded and then averaged to measure PV and tPV, respectively. As a measure of movement efficiency, we calculated OE at each joint, using the difference between a subject’s maximum extension and final position, to determine if a joint was overextended.

In Phase 4, subject’s performed tasks that were previously un-encountered. To determine if synergy postures were being incorporated into these movements, the final posture from each task was reconstructed using subject-specific synergies. The RE was measured as the Euclidian error between actual and reconstructed postures, summed across the eight joints.

Finally, spatial trends were evaluated using the position of each button. Tasks that involved a specific button (1 of the 12) were first grouped. The percentage of tasks correct in this group was measured for each subject. This was repeated for all 12 buttons.

First, to verify parity across the three groups during Phase 1 (preliminary evaluation), a one-way analysis of variance (ANOVA) test for PC, CT, RT, PV, tPV, and OE was performed. We found that group means obscured individual subject changes. Thus, to offset subject differences, we first measured how a variable changed between phases. Accordingly, rather than using a two-way repeated measures ANOVA, a one-way ANOVA across groups was used for each phase. Multiple comparisons were used to test significant results using a Tukey–Kramer test (*p* < 0.05). For variables specific to Phase 4 (RE, button-specific performance), one-way ANOVA were used to detect differences across groups. Because of non-normal distribution in RE, even after log transformation, a non-parametric Kruskal–Wallis test was also performed. In all tests, significance was set to *p* < 0.05.

## Results

### Task Performance and Kinematics

**Figure 5:**
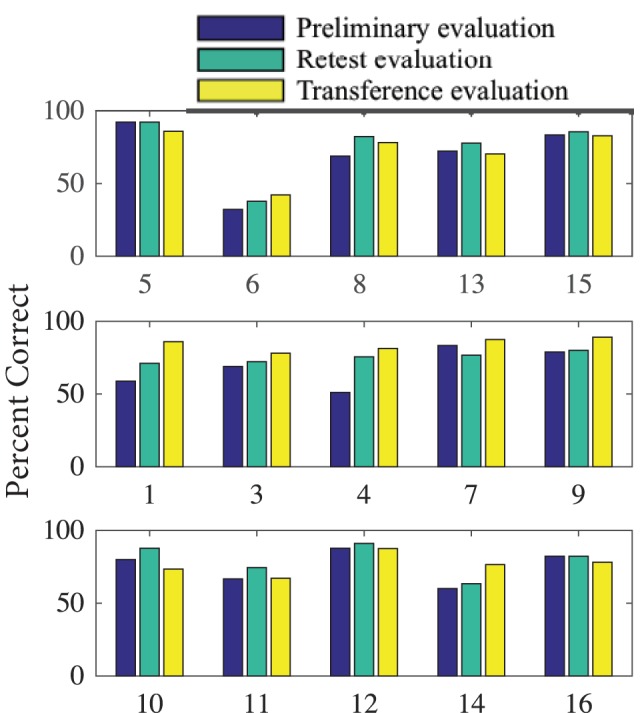
**Percent of tasks correct for each subject during Phase 1 (preliminary evaluation), Phase 2 (retest evaluation), and Phase 4 (transference evaluation)**. Subjects are organized by their training (task repetition, synergy repetition, and control) group.

Results for task performance and kinematic measures are presented in Table 1. Across all phases and groups, subjects’ scores for percent correct (PC) ranged from 32 to 92%. Figure 5, however, shows an outlier subject (subject 6), while all other performance scores were similar. CT averaged ~2 s, of which the first ~0.29 s was RT. tPV and time of peak extension (tPE) occurred at ~0.65 and ~1.14 s, respectively. Average OE across joints was ~6° but ranged from 0° to 60°.

**Table 1:** **Group averages ± standard deviation for task performance and kinematic measures during Phase 1, Phase 3, and Phase 4**.

	Task repetition group			Synergy repetition group			Control group		
Phase #	1	3	4	1	3	4	1	3	4
PC (%)	69.778 ± 22.94	75.111 ± 21.52	71.875 ± 17.608	68.222 ± 13.44	75.111 ± 3.57	84.375 ± 4.55	75.333 ± 11.56	79.778 ± 11.15	76.563 ± 7.412
CT (s)	2.225 ± 0.0261	2.1159 ± 0.365	2.21551 ± 0.3566	2.096 ± 0.0601	1.9760 ± 0.565	2.0304 ± 0.624	2.082 ± 0.0206	2.1240 ± 0.263	2.1267 ± 0.3069
RT (s)	0.293 ± 0.06	0.285 ± 0.0288	0.303905 ± 0.054	0.368 ± 0.14	0.310 ± 0.0879	0.277 ± 0.07	0.254 ± 0.05	0.232 ± 0.0575	0.258 ± 0.053
PV (°/s)	234.04 ± 36.43	210.89 ± 41.88	221.3861 ± 53.38	237.82 ± 59.52	241.39 ± 39.55	235.12 ± 30.49	244.45 ± 22.02	230.58 ± 23.153	254.89 ± 15.39
tPV (s)	0.653 ± 0.09	0.640 ± 0.0471	0.687102 ± 0.087	0.753 ± 0.27	0.716 ± 0.2717	0.655 ± 0.174	0.588 ± 0.05	0.580 ± 0.0872	0.594 ± 0.106
tPE (s)	1.360 ± 0.09	1.299 ± 0.1603	0.869646 ± 0.0985	1.315 ± 0.30	1.199 ± 0.305	0.819 ± 0.211	1.208 ± 0.12	1.260 ± 0.1795	0.871 ± 0.180
OE (°)	6.556 ± 8.86	5.42 ± 7.79	6.29 ± 8.5	6.172 ± 9.4743	8.068 ± 13.039	8.94 ± 12.28	6.705 ± 8.853	6.086 ± 8.533	6.3 ± 9.00

*PC, percent correct; CT, completion time; RT, reaction time; PV, peak velocity; tPV, time of peak velocity; tPE, time of peak extension; OE, overextension*.

*Means and 1 SD are given*.

Phase 1 (preliminary evaluation) measurements were tested using a one-way ANOVA to ensure parity across the groups. Table 2 (column 2) shows that none of the variables showed significant difference across groups. However, as seen in Table 1, within-group SDs for all measured variables are relatively large. Thus, to reduce the effect of intersubject differences, we calculated how measurements for each variable changed across phases. Table 2 shows group averages for these changes from Phase 1 to Phase 3 (retest evaluation) and from Phase 3 to Phase 4 (transference evaluation). ANOVA results showed a significant group difference in tPE after training (*p* = 0.034). Subjects in both the task repetition group and synergy group were able to reach peak extension faster after training. However, the control group averaged an increase in tPE. Multiple comparisons show only a significant difference between the synergy group and control group (*p* = 0.030). A significant group difference was found for changes in PC from Phase 3 to Phase 4 (*p* = 0.019). When tested on the second set of tasks in Phase 4, subjects in the synergy training group were able to improve their performance while subjects in the task repetition training group and control group showed decreased performance. This is more clearly shown in Figure 5, which provides PC for each subject in different phases. Multiple comparisons showed that the average PC change for the synergy group was significantly greater than that of the task repetition group (*p* = 0.033) and control group (*p* = 0.033). A significant group difference was also found for PV. Subjects in the control group expressed an average increase of 24.3 ± 19.71 /s in PV. This was significantly different than synergy group’s average decrease of 6.27 ± 16.73 /s (*p* = 0.037). No clear trends or significant differences were observed for CT, RT, and OE.

**Table 2:** **One-way analysis of variance results for each variable during each phase**.

	*p* Value	Phase 3–Phase 1			*p* Value	Phase 4–Phase 3			*p* Value
		TR	SR	C		R	SR	C	
PC (%)	0.836	5.3 ± 5.1	6.9 ± 11.9	4.5 ± 3.3	0.880	−3.2 ± 4.7	9.3 ± 3.8	−3.2 ± 10.1	**0.019**
CT (s)	0.822	−0.1094 ± 0.1598	−0.1199 ± 0.1385	0.0411 ± 0.2583	0.367	0.0996 ± 0.1070	0.0544 ± 0.2211	0.0028 ± 0.1236	0.639
RT (s)	0.178	−0.008 ± 0.0487	−0.058 ± 0.0773	−0.021 ± 0.039	0.391	0.0191 ± 0.0394	−0.0327 ± 0.0309	0.0262 ± 0.0489	0.079
PV (°/s)	0.421	−23.14 ± 29.10	3.57 ± 23.15	−13.87 ± 22.88	0.274	10.50 ± 14.06	−6.27 ± 16.73	24.30 ± 19.71	**0.045**
tPV (s)	0.461	−0.0125 ± 0.0668	−0.0372 ± 0.0221	−0.0077 ± 0.0679	0.684	0.0471 ± 0.0714	−0.0609 ± 0.1051	0.0140 ± 0.0421	0.684
tPE (s)	0.782	−0.0612 ± 0.0968	−0.1162 ± 0.1025	0.0520 ± 0.0667	**0.034**	−0.4294 ± 0.1425	−0.3801 ± 0.1419	−0.3891 ± 0.1070	0.822
OE (°)	0.357	−1.1 ± 0.7	1.8 ± 3.6	−0.4 ± 1.6	0.154	−1.6 ± 0.8	−2.2 ± 1.9	−2.0 ± 1.0	0.752

*Column 2 *p* values show that no significant differences between groups were found for any variables during Phase 1 (group averages are provided in Table 1). Changes from Phase 1 to Phase 3 and from Phase 3 to Phase 4 are presented in columns 3–5 and 7–9 for each group: task repetition training (TR), synergy repetition training (SR), and control (C)*.

*PC, percent correct; CT, completion time; RT, reaction time; PV, peak velocity; tPV, time of peak velocity; tPE, time of peak extension; OE, overextension*.

*Significant group differences are indicated in bold *(p* < 0.05)*.

**Figure 6:**
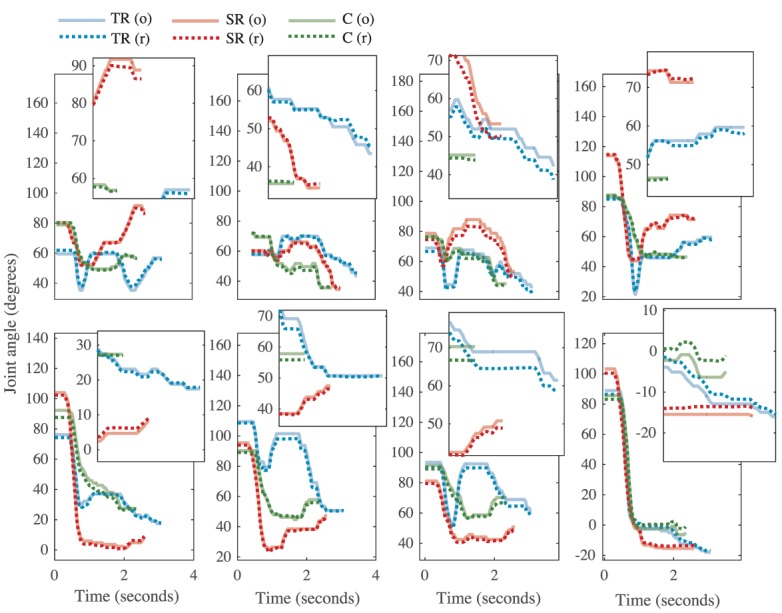
**The kinematic profile for task # 26 from representative subjects from each group [task repetition (TR)-blue, synergy repetition (SR)-red, and control (C)-green]**. Metacarpophalangeal (MCP) joints and proximal interphalangeal (PIP) joints are shown in the top and bottom rows, respectively. Finger abbreviations are index (I), middle (M), ring (R), and pinky (P). Each joint profile has an inset that magnifies the profile at the end of the task, where differences between original (o, solid lines) and reconstructed (r, dotted lines) can be better appreciated.

**Figure 7:**
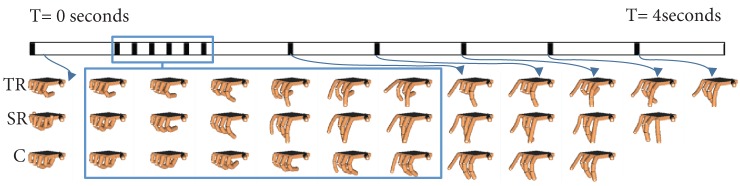
**Task # 26 from Phase 4 is shown**. A single subject from the task repetition group (TR), synergy repetition group (SR), and control group (C) is used as representative example. A time (*T*) bar is provided to show how the task unfolds from the beginning of the task (*T* = 0 s) to the end of the task (*T* = 4 s). Blank hand spaces indicate that the task was completed early. Joint based analysis showed that the task repetition group overextended metacarpophalangeal joints to a greater extent than synergy repetition and control groups.

Because many of the kinematic variables are averaged across joints, we further explored how the four fingers individually executed the tasks. A temporal analysis revealed interesting trends that spanned all three groups. Figures 6 and 7 show how the same task (task # 26 from Phase 4) was executed by a representative subject from the task repetition group, synergy group, and control group. In Figure 6, profiles for each of the eight recorded joints are presented and are overlaid by their reconstructed versions. In general, index and pinky MCP joints averaged the fastest times to reach peak extension, but their PIP joint extensions occurred last. The index MCP joint also had exhibited the most OE for all three groups. Time to reach peak extension in the middle and ring PIP joints averaged similar times. For each joint, we separately recomputed time to peak extension and OE. Between-group ANOVA results are provided in Table 3. At all four MCP joints, the task repetition group showed significantly greater OE than the synergy group and control group. However, M_MCP, R_MCP, P_MCP, I_PIP, and M_PIP joints in the task repetition group reached peak extensions significantly faster than synergy and control groups (*p* < 0.05).

**Table 3:** **Results of joint-specific analysis for overextension (OE) measurements and time to peak extension measurements (s)**.

	OE (°)			*p* Values	Time to peak extension (s)			*p* Values
	TR	SR	C		TR	SR	C	
I_MCP	**15.01^a^ ± 13.76**	11.23 ± 9.77	11.95 ± 11.08	**0.0006**	0.9412 ± 0.473	1.0125 ± 0.448	0.9476 ± 0.475	0.1803
M_MCP	**6.90^b^ ± 9.82**	4.38 ± 6.01	5.87 ± 6.94	**0.0017**	**1.1311^b^ ± 0.750**	1.2890 ± 0.637	1.2171 ± 0.740	**0.0471**
R_MCP	**9.12^a^ ± 11.66**	3.72 ± 5.62	2.99 ± 5.78	**<0.0001**	**1.1909^a^ ± 0.681**	1.5527 ± 0.679	1.5631 ± 0.705	**<0.0001**
P_MCP	**15.29^a^ ± 18.23**	6.42 ± 8.28	4.14 ± 6.40	**<0.0001**	**1.0057^a^ ± 0.534**	1.2693 ± 0.599	1.2443 ± 0.562	**<0.0001**
I_PIP	**3.27^c^ ± 5.59**	**3.25^c^ ± 6.83**	1.87 ± 4.31	**0.0072**	**1.5080^a^ ± 0.701**	1.6396 ± 0.511	1.6657 ± 0.604	**0.0078**
M_PIP	9.72 ± 11.07	7.88 ± 9.11	9.62 ± 11.70	0.1075	**1.1500^a^ ± 0.629**	1.2983 ± 0.528	1.2857 ± 0.513	**0.0043**
R_PIP	8.62 ± 10.62	9.26 ± 20.24	10.64 ± 10.70	0.0914	1.2016 ± 0.561	1.2690 ± 0.511	1.1999 ± 0.528	0.2747
P_PIP	3.63 ± 6.82	4.14 ± 7.17	3.18 ± 5.00	0.2669	1.5934 ± 0.696	1.6204 ± 0.642	1.5909 ± 0.655	0.8670

*Post hoc significant results are bolded*.

*^a^A significant difference between task repetition and synergy repetition groups and between task repetition and control groups*.

*^b^Significant difference between the task repetition and synergy repetition groups*.

*^c^Significant difference between task repetition and control groups and between synergy repetition and control groups*.

*TR, task repetition; SR, synergy repetition; C, control; MCP, metacarpophalangeal; PIP, proximal interphalangeal; I, index; M, middle; R, ring; P, pinky*.

### Utilization of Synergies

**Figure 8:**
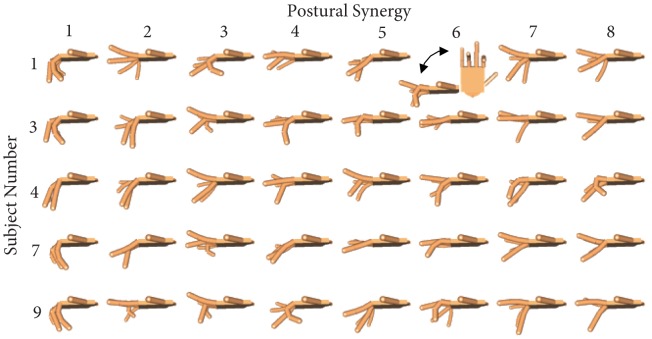
**For each subject in the synergy repetition group, eight postural synergies were derived and used for training in Phase 2**. Postural synergy 1 accounts for the most variance and is characterized by general flexion in all joints. Postural synergies 2 through 8 account for decreasing amount of variance from the dataset but still represent used joint coordination patterns.

Subjects in the synergy training group were each trained on eight distinct postural synergies. These postures are presented in Figure 8. The first synergy is similar across all subjects and is characterized by MCP and PIP flexion, albeit at different magnitudes. The remaining postures emphasize alternating relationships among the fingers. Importantly, difficult and less commonly used postures have been captured by these synergies. For example, synergy 6 from subject 1 shows the ring PIP joint flexion while the pinky MPC and PIP joints are extended. Because the pinky is partly enslaved to the ring finger, this posture requires conscientious digression from natural behaviors.

**Figure 9:**
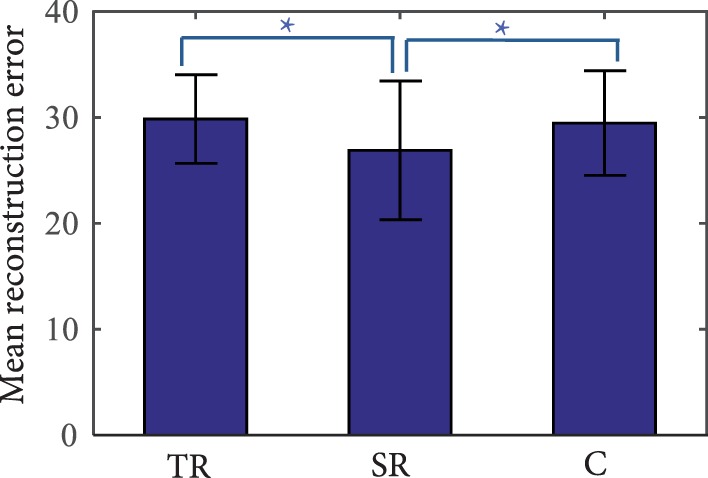
**Reconstruction error (RE) of tasks in Phase 4 (transference evaluation)**. The synergy repetition group (SR) has significantly lower RE than the task repetition (TR) and control (C) groups as indicated by the *p less than or equal to 0.001.

Results from the task performance analysis show that the synergy group performed significantly better in the Phase 4 (transference evaluation) compared to the task repetition and control groups. Thus, the next step is to determine how well synergies were being incorporated into these movements. Correct tasks in Phase 4 were reconstructed using a subject’s corresponding synergies. For comparison, synergies of the task repetition and control groups were also derived and used to reconstruct end postures. An example reconstruction of each joint was provided in Figure 6. RE is used as a measure of synergy usage and is presented in Figure 9. Note that only the error from the end posture, and not the entire time profile was used. Results show that end postures in the synergy group were reconstructed with significantly less error than both the repetition group (ANOVA, *p* = 3.8e−9; Kruskal–Wallis, *p* = 4.45e−6) and control group (ANOVA, *p* = 1.83e−7; Kruskal–Wallis, *p* = 0.001). These results show that postures used during Phase 4 more closely resembled synergies in the synergy group, than in the repetition and control groups.

### Task Analysis

**Figure 10:**
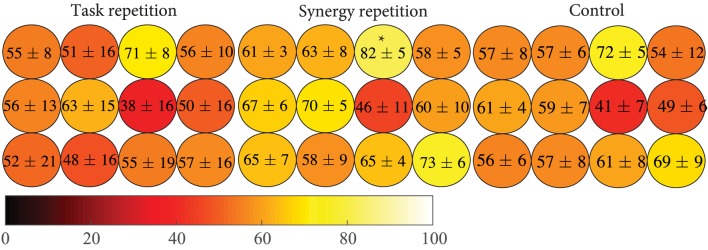
**Individual button-based performance was calculated for each group**. The color scale indicates the percentage of tasks correct with lighter colors representing better performance. The synergy repetition group shows better performance overall but only was only significantly greater than the task repetition group (*p* = 0.032) at ring, maximum extension (indicated by the *).

Results indicate that subjects in the synergy group were able to employ postures that they were trained on. These practiced postures possibly led to the performance improvements previously described. To further explore the benefits of practicing these different hand configurations and how they may have been incorporated into the task, a spatial analysis was used to evaluate the performance of different fingers. Phase 4 tasks that involved a specific button were grouped. Then, the performance of each group across these tasks was measured. Results are shown in Figure 10. The distribution of performance in all 12 buttons was similar across the three groups. Specifically, all three groups showed the worst performance when moderate extension was required of ring MCP and PIP joints (third column, middle row), but best performance when maximum extension was required of ring MCP and PIP joints (third column, top row). ANOVA results showed a significant group difference at this location (*p* = 0.0252). The synergy group was able to perform these tasks significantly better than the task repetition group (*p* = 0.032) but did not reach significance for the control groups (*p* = 0.058). All other button locations show that the synergy group had distributed advantages.

## Discussion

Motor learning is often characterized by acquisition, retention, and transference of a new motor skill (Magill and Anderson, 2007). Quantitatively, motor learning exhibits as improvements in time and accuracy. In this study, we compared two different training groups, task repetition (gold standard) and synergy training, to assess differences in motor learning. Our results (summarized in Table 4) show that when the same tasks were tested, both, task repetition and synergy training group, showed greater improvements (increased percent correct and faster time to reach peak extension) than the control group (Tables 1 and 2). We expected subjects in the task repetition group to perform better than subjects in the synergy group during Phase 3 (retesting). Significant advantage was only demonstrated when excluding subject 6, who had outlier (third SD) performance in PC. Regardless, subjects from both these groups, at least for short term, were able to retain learned task dynamics. However, when tested on the new task, only subjects in the synergy training group were able to transfer their new motor skill, as evidenced by continued improvement in accuracy. The better performance was not concentrated by a single finger or extension level but spanned all 12 buttons as seen in Figure 10. It is worth noting that the button analysis showed that buttons 5 and 12 (see Figure 4) were used more often in set 1 tasks and buttons 6 and 11 were used more often in set 2 tasks. Figure 10 shows that all groups may have been affected by being tested on buttons that they were less exposed to during training (although the control group shows the same trend). In other words, button 6 and 11 locations showed lower averages compared to other buttons. However, it is unclear if this resulted from limited pre-valuation/training exposure or that the button location themselves were more difficult. Next, we discuss how both training methods may have influenced motor learning.

**Table 4:** **Overall results to compare task repetition training and synergy repetition training**.

	Task repetition training	Synergy repetition training
Training protocol	45 tasks, repeated twice	10 synergy postures, each repeated 5–6 times
Training protocol strengths	More time spent with test environment Hand configuration directly related to task goal Greater number of tasks	Equal time spent with difficult hand patterns as well as common, easier hand patterns Concentration on a few key postures
Approximate number of postures performed in 20 min training period	45 postures, with two repetitions	~10 postures, each with 5–6 repetitions
Average performance change in Phase 3 (retest)	5.3% ± 5.1%	6.9% ± 11.9%
Average performance change in Phase 4 (transference)	−3.2% ± 4.7%	9.3% ± 3.8%
Kinematic changes in Phase 3	Peak extension reached faster	Peak extension reached faster
Kinematic changes in Phase 4	Greater over extension, Peak extension reached faster compared to other groups	Decrease in peak velocity Overall, greater target button hit rate

In our study, numerous explicit learning mechanisms were implemented. For example, if a task was performed correctly, a “correct indicator” was lit green. Subjects were verbally told to accomplish tasks simultaneously across all fingers. Additionally, subjects familiarized themselves with start and stop cues so that the task could be accomplished within the allotted time. These factors contribute to explicit learning using external cues provided by the experimenter. Subjects in the task repetition group were more exposed to explicit mechanisms because their training phase provided more time in the task environment. The extra allotted time also allowed cognitive processes to create an optimal motor plan for each task. Subjects in the synergy training group, however, were trained using different explicit mechanisms, synergies.

The development of hand synergies, as well as other motor synergies (i.e., walking), begins early on when infants use imitation and/or self-regulated learning mechanisms to achieve a grasp (Oztop et al., 2004). Between 9 and 13 months, reach and grasp motor patterns appear pre-programmed, evidenced by temporal overlap and early anticipatory movements (Lockman et al., 1984; Newell et al., 1993). Konczak et al. (1995) found that a “fine tuning” period in infants 7–18 months was characterized by stable joint torque patterns over time, and across limb segments. Further analysis of shoulder, arm, and hand kinematics showed that these infants express stereotypical kinematic patterns only after 24 months (Konczak and Dichgans, 1997). Through mainly implicit learning mechanisms, these infants stored the most effective and common motor sequences required for grasping, optimizing them throughout life. Computationally, we derive these synergies through dimensionality reduction techniques, which capture primed inter-joint coordination. In this study, a new touch task required subjects to develop new inter-joint coordination techniques in order to complete the tasks correctly and quickly. All three groups may have implicitly learned useful inter-joint coordination during the initial evaluation (Phase 1) and retest (Phase 3). However, subjects in the synergy group received more concentrated training on these inter-joint coordination patterns through postural synergy training. The reconstruction analysis (Figure 5) indicates that these trained postures were used during the transference tests. Additionally, kinematic analysis indicates that synergy training affected the feed-forward mechanisms (resulting from motor planning) allowing joints to reach their most extended configurations, quicker. In the task repetition group, joints were extended quicker, but this also caused significantly greater OEs in Phase 4.

Although only healthy adults were used in this study, our results show that synergy training may be able to address some of the requirements of poststroke physical therapy. For example, the question of whether therapy should be constant versus variable has been addressed in numerous studies (Lin et al., 2008; Wu et al., 2011). While constant, repetitive practice reinforces positive mechanisms, it may reduce the ability to transfer a skill (Dromerick et al., 2009) potentially because of less exposure to all the inherent task patterns (Meier and Cock, 2010). Concurrently, the synergy group concentrates only these patterns. Additionally, equal training time is given to all patterns, whether common or not. This type of training resulted in spatially broad advantages (Figure 10) during the transference tasks. However, intense repetition training also leads to improvements in function after stroke (Kawahira et al., 2004). Thus, a balance between repetition and synergy training may provide optimal results. Additionally, the implications of “whole versus part” training in neurorehabilitation have been explored (Schmidt and Lee, 2011; Wickens et al., 2013). For example, in relearning “reach and grasp,” poststroke, it is important to maintain the overlapping temporal relationship between arm transport and hand grasp. While synergies derived in this study were static postures, training with spatio-temporal synergies (Vinjamuri et al., 2010) would allow individuals to reinforce temporal relationships. Finally, evidence suggests that in adults with neurological damage in certain brain areas, explicit instructions can lead to poorer performance than implicit instructions (Boyd and Winstein, 2003), suggesting that rehabilitation efforts need to balance how supposed explicit and implicit knowledge can be delivered. This balance may be reached with the use of synergies.

While this study attempted to model the benefits of synergy-based learning for potential use in motor learning as well as hand rehabilitation, there are some limitations to consider. First, the sample size in this data set is quite small. Based on results of the main outcome measure of this study (PC in Phase 4), we would need a sample size of 20 subjects in each group for 80% power (α = 0.05). Thus, the current low sample size of *n* = 5 in each group only achieves has an extremely low power and thus, high type II (false negative) error rate. A significant group difference was indeed found (Cohen’s *d* = 0.401, moderate effect), but only after accounting for intersubject differences. Future studies may have to establish a more equivalent baseline with lower SDs. Second, synergies that were used for training were not necessarily optimal because they were acquired relatively early in training stages. Moreover, they were subject specific. In a realistic setting, synergies need to be derived from healthy, skilled individuals to be used on unskilled individuals. Minor unnatural discrepancies between individuals may cause undue difficulty in training. Third, the task used in this study was created to balance novelty and finger range of movement. Other hand-related motor tasks, such as the serial RT task (Robertson, 2007), have been extensively researched in their ability to expose different motor-learning strategies. In designing the task for this study, we attempted to balance novelty with hand-related function. Further investigation is required to delineate the explicit mechanisms that may have occurred during the task and also determine their effects on long-term retention of the skill.

## Ethics Statement

The study was approved by IRB at Stevens Institute of Technology. Consent procedures were followed according to the IRB guidelines at Stevens. All subjects were individuals without any disabilities.

## Author Contributions

Methods and experimental setup were designed and implemented by VP and JC, with guidance from RV. MS helped with data collection and data analysis. MB helped create data collection program. IF helped with statistics. Manuscript was written by VP and revised and approved by all authors.

## Conflict of Interest Statement

The authors declare that the research was conducted in the absence of any commercial or financial relationships that could be construed as a potential conflict of interest.
